# CT radiomics model for predicting the Ki-67 proliferation index of pure-solid non-small cell lung cancer: a multicenter study

**DOI:** 10.3389/fonc.2023.1175010

**Published:** 2023-08-29

**Authors:** Fen Liu, Qingcheng Li, Zhiqiang Xiang, Xiaofang Li, Fangting Li, Yingqiong Huang, Ye Zeng, Huashan Lin, Xiangjun Fang, Qinglai Yang

**Affiliations:** ^1^ Department of Radiology, The Second Affiliated Hospital, Hengyang Medical School, University of South China, Hengyang, China; ^2^ Department of Radiology, The Affiliated Huaihua Hospital, Hengyang Medical School, University of South China, Huaihua, China; ^3^ Department of Radiology, People’s Hospital of Zhengzhou, Zhengzhou, China; ^4^ Department of Radiology, The Second Affiliated Hospital of Hainan Medical University, Haikou, China; ^5^ Department of Radiology, The First Affiliated Hospital, Hengyang Medical School, University of South China, Hengyang, China; ^6^ Department of Pharmaceutical Diagnosis, GE Healthcare, Changsha, China; ^7^ Center for Molecular Imaging Probe, Hunan Province Key Laboratory of Tumor Cellular and Molecular Pathology, Cancer Research Institute, Hengyang Medical School, University of South China, Hengyang, Hunan, China

**Keywords:** radiomics, Ki-67, nomogram, non-small cell lung cancer, multicenter study

## Abstract

**Purpose:**

This study aimed to explore the efficacy of the computed tomography (CT) radiomics model for predicting the Ki-67 proliferation index (PI) of pure-solid non-small cell lung cancer (NSCLC).

**Materials and methods:**

This retrospective study included pure-solid NSCLC patients from five centers. The radiomics features were extracted from thin-slice, non-enhanced CT images of the chest. The minimum redundancy maximum relevance (mRMR) and least absolute shrinkage and selection operator (LASSO) were used to reduce and select radiomics features. Logistic regression analysis was employed to build predictive models to determine Ki-67-high and Ki-67-low expression levels. Three prediction models were established: the clinical model, the radiomics model, and the nomogram model combining the radiomics signature and clinical features. The prediction efficiency of different models was evaluated using the area under the curve (AUC).

**Results:**

A total of 211 NSCLC patients with pure-solid nodules or masses were included in the study (N=117 for the training cohort, N=49 for the internal validation cohort, and N=45 for the external validation cohort). The AUC values for the clinical models in the training, internal validation, and external validation cohorts were 0.73 (95% CI: 0.64–0.82), 0.75 (95% CI:0.62–0.89), and 0.72 (95% CI: 0.57–0.86), respectively. The radiomics models showed good predictive ability in diagnosing Ki-67 expression levels in the training cohort (AUC, 0.81 [95% CI: 0.73-0.89]), internal validation cohort (AUC, 0.81 [95% CI: 0.69-0.93]) and external validation cohort (AUC, 0.78 [95% CI: 0.64-0.91]). Compared to the clinical and radiomics models, the nomogram combining both radiomics signatures and clinical features had relatively better diagnostic performance in all three cohorts, with the AUC of 0.83 (95% CI: 0.76–0.90), 0.83 (95% CI: 0.71–0.94), and 0.81 (95% CI: 0.68–0.93), respectively.

**Conclusion:**

The nomogram combining the radiomics signature and clinical features may be a potential non-invasive method for predicting Ki-67 expression levels in patients with pure-solid NSCLC.

## 1 Introduction

Non-small cell lung cancer (NSCLC) is the most common pathological type of lung cancer, accounting for more than 85% of cases (1). On multi-slice spiral computed tomography (MSCT) images, early NSCLC may present as two subtypes: pure-solid or subsolid nodules (2). These two radiological subtypes of lung cancer may have different biological behaviors. NSCLC pure-solid nodules or masses usually exhibit more aggressive malignant behavior, while patients presenting with such radiological subtypes usually have a worse prognosis (3).

The treatment choice for NSCLC heavily relies on molecular biomarkers (4). Ki-67 PI is the most commonly used marker to assess the proliferation of tumor cells, and its expression closely correlates with tumor metastasis and poor prognosis (5). In lung cancer, expression levels of Ki-67 can indirectly indicate tumor invasion (1). According to a recent study, more than 90% of oncologists believe that lung cancer treatment might depend on Ki-67 expression (6).

Surgical and puncture biopsy samples are the gold standard for determining intratumoral Ki-67 expression levels. However, some tumors are inaccessible for biopsy collection, while certain patients cannot tolerate invasive examinations. Thus, finding a simple and non-invasive method that accurately predicts Ki-67 expression is an unmet clinical need.

Radiomics uses high-throughput technology to extract quantitative information from radiographic images, providing more clinically relevant information than traditional imaging analysis (7). Radiomics has demonstrated outstanding potential for lung cancer diagnosis, tumor classification, prognosis prediction, and tumor gene expression analysis (8–11). Ki-67 expression levels can also be predicted by radiomics, as shown in previous studies (8, 12–14). However, since pure-solid and subsolid NSCLC may have very different biological characteristics (3), the ability of radiomics to predict Ki-67 expression in pure-solid and subsolid NSCLC may also be different. To the best of our knowledge, few multicenter studies investigate whether radiomics can predict the expression level of Ki-67 in NSCLC of pure-solid nodules or masses. To address this knowledge gap, we performed a retrospective multicenter study to construct and validate a CT radiomics model for predicting Ki-67 expression in patients with pure-solid NSCLC.

## 2 Materials and methods

### 2.1 Patient selection

This study was approved by the institutional ethics committee of the Second Affiliated Hospital, Hengyang Medical School, University of South China. A total of 211 patients who underwent surgical resection or puncture biopsy between January 2018 and June 2022 were enrolled from five centers (center 1:The Second Affiliated Hospital, Hengyang Medical School, University of South China; center 2:The First Affiliated Hospital, Hengyang Medical School, University of South China; center 3:The Affiliated Huaihua Hospital, Hengyang Medical School, University of South China; center 4: People’s Hospital of Zhengzhou; center 5:The Second Affiliated Hospital of Hainan Medical University).

The inclusion criteria were as follows: (1) Patients with NSCLC confirmed by puncture biopsy or surgical pathology with complete DICOM format CT thin-section images, Ki-67 test results, and clinicopathological data; (2) Puncture biopsy or surgical resection within one month after the thin-section CT examination; (3) The tumors presenting with pure-solid nodules or masses.

The exclusion criteria were as follows: (1) Unclear lesion boundary preventing accurate tumor outline; (2) Treatment or an invasive examination before CT examination; (3) Incomplete clinical data and/or pathological results; (4) Tumors contained ground-glass components; (5) History of other primary tumors unrelated to NSCLC.

**Figure 1:**
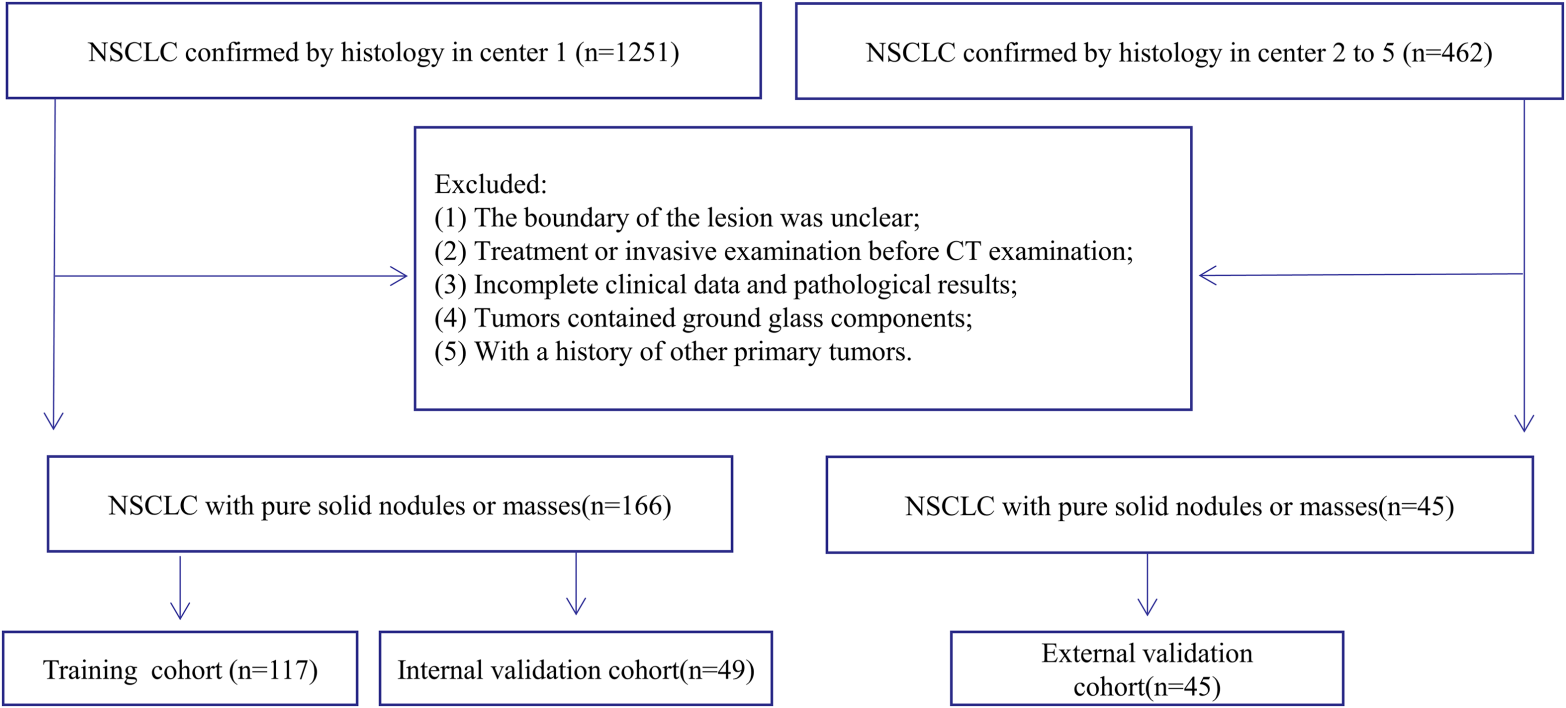
Flowchart showing the selection process of study subjects.

A total of 211 patients were enrolled in this study, including 166 patients from Center 1 and 45 from Centers 2 to 5 (**Figure 1**). The patients with pure-solid nodules or masses from center 1 were randomly assigned to the training and internal validation cohorts at a 7:3 ratio; patients from centers 2 to 5 were assigned to an external validation cohort.

### 2.2 CT scanning and image acquisition

Spiral non-enhanced CT scans of the chest were performed on all patients participating in the study. **Table 1** shows the details of the CT scanning routine and image reconstruction for each hospital participating in the study. The scanning range covered the chest entrance to the bilateral adrenal level.

**Table 1:** CT scanning parameters for each hospital participating in the study.

	Center 1		Center 2	Center 3	Center 4	Center 5
	CT64	CT128	CT64	CT64	CT64	CT64
Manufacturer	Philips Brilliance 64	Philips iCT 256	Siemens Somatom Definition AS	Siemens Somatom Definition Flash	Siemens Definition	Siemens Somatom Definition Flash
Convolution kernel	E/F/L	B/E/L	B40f	I70f\3	B30f	I70f\3
Tube voltage (kV)	120	120	120	120	120	120
Tube current(mA)	100-250	100-250	Auto	100-400	200-300	100-300
Matrix	512×512	512×512	512×512	512×512	512×512	512×512
Slice thickness (mm)	5	5	5	5	3	5
Reconstructed slice thickness	1	1	1	1	1	1
CDTIvol (mGy)	4.5-13.1	4.0-15.1	7.2-15.2	5.2-12.3	5.8-12.5	4.3-13.2

### 2.3 Ki-67 immunoassay

A monoclonal mouse anti-human Ki-67 antibody was used in this study. The detection of Ki-67 was performed per the manufacturer’s instructions. For each glass slide, 1000 cells were randomly selected, and positive cells were counted. In accordance with previous studies, <40% positivity was defined as low Ki-67 expression, while >=40% positivity was defined as high Ki-67 expression (13, 15, 16).

### 2.4 Evaluation of clinical information

In this study, all the CT images were independently evaluated by two diagnostic radiologists with more than three years of experience in standard lung windows (window width 1600 HU; window position - 600 HU). A third radiologist with ten years of experience resolved any disagreements. The following clinical information was evaluated: age, gender, smoking status, tumor pathological type, Ki-67 expression, and CT morphological and semantic features.

### 2.5 Tumor segmentation and feature extraction

**Figure 2:**
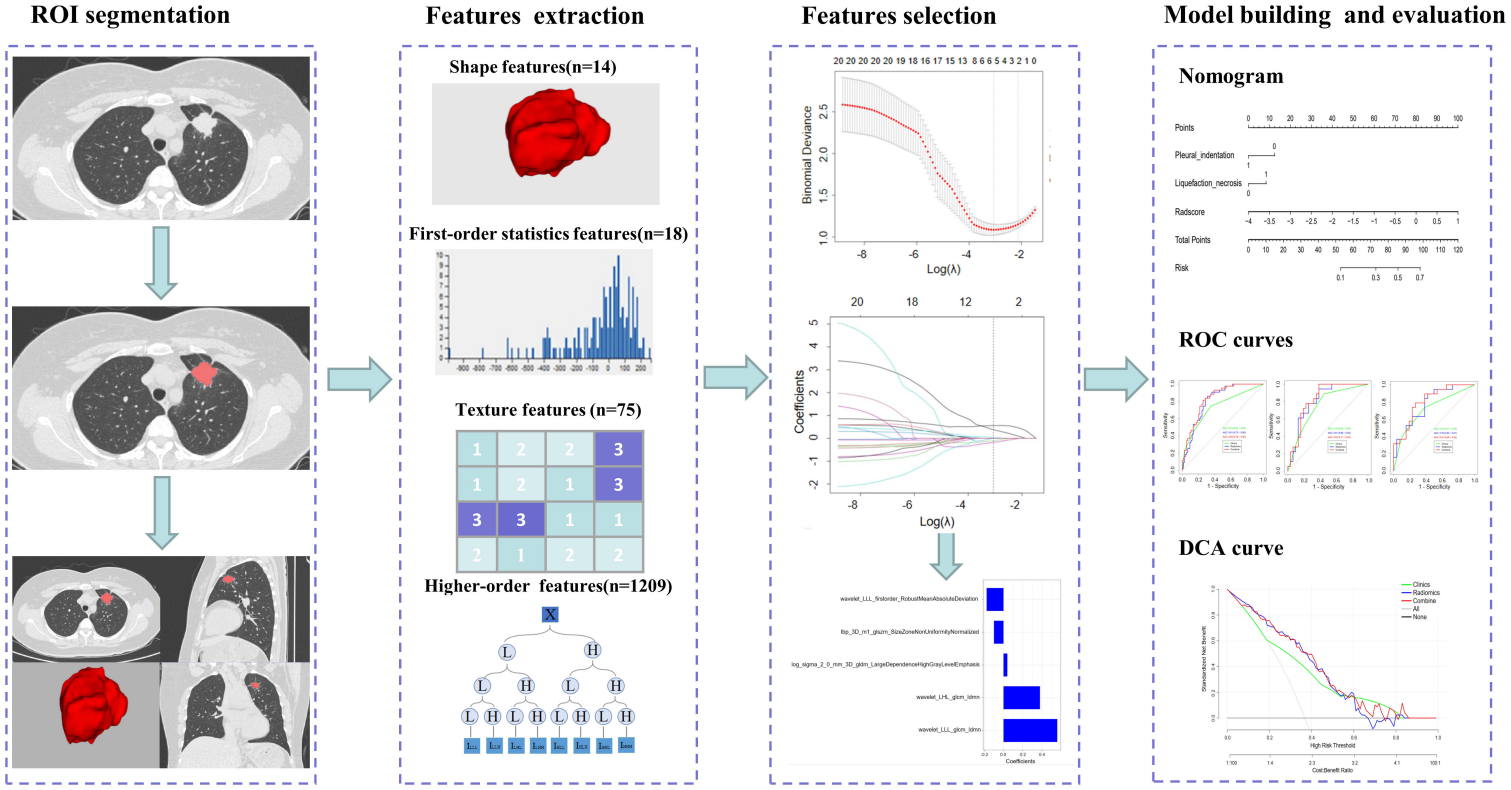
Overview of the radiomics workflow.

Using the ITK-SNAP software (version 3.8.0; www.itksnap.org), each DICOM image’s region of interest (ROI) was manually delineated in a 3D space. All ROIs were then outlined by two radiologists with more than three years of experience; all the lesions were labeled layer by layer, and the tumor boundaries were outlined to exclude non-tumor structures such as blood vessels, bronchi, and pleura. All the outlined ROIs were imported into the AK software (Analysis Kit, GE Healthcare, Chicago, IL, USA). The images were processed by pixel size normalization and resampling to extract the radiomics features. Altogether, 1316 features were extracted, including 18 first-order statistical features, 14 shape features, 75 texture features, and 1209 higher-order features. The workflow for radiomics is shown in **Figure 2**. The intraclass correlation coefficient (ICC) was used to analyze the consistency of the image features extracted by the radiologists.

### 2.6 Selecting features and building the radiomics signature

The minimum redundancy maximum relevance (mRMR), and least absolute shrinkage and selection operator (LASSO) were used to select the optimal radiomics features. The mRMR was used to eliminate redundant and irrelevant features. Subsequently, the LASSO was conducted to select the optimized subset to construct the radiomics score (rad-score). The rad-score calculation formula was obtained using the characteristics and their coefficients, followed by the computation of the rad-score value.

### 2.7 Establishment of prediction models

A Logistic regression model was developed based on the univariable and multivariable analyses of clinical characteristics. Three different prediction models were established to predict the diagnostic efficacy of Ki-67 PI: the clinical model, the radiomics model, and the nomogram combining radiomics signature and clinical features.

### 2.8 Statistical analysis

The R software version 4.0.2 and SPSS version 26.0 were used to analyze data. The prediction models were tested using data from internal and external validation cohorts. Univariable and multivariable analyses were used for clinical feature selection. The receiver operating curve (ROC) and AUC were used to evaluate the diagnostic efficacy of the model. Decision curve analysis (DCA) was employed to evaluate the clinical usefulness of three prediction models. Mann-Whitney-Wilcoxon test was used to compare continuous variables, the chi-squared test was used for categorical variables, and *P*<0.05 indicated that the difference was statistically significant.

## 3 Results

### 3.1 Clinical characteristics of the three cohorts in the Ki-67-high and Ki-67-low expression groups

A total of 211 patients were included in this study. The detailed clinical characteristics of the three cohorts are presented in **Table 2**. Among the most significantly different clinical characteristics in the Ki-67-high group versus the Ki-67-low group were the pathological type, longest diameter, spiculation sign, pleural indentation, and liquefaction necrosis (*P*<0.05).

**Table 2:** The clinical characteristics of three cohorts.

Clinical characteristics	Training cohort (n=117)		*P* -value	Internal validation cohort (n=49)		*P -*value	External validation cohort (n=45)		*P* -value
	Low Ki-67	High Ki-67		Low Ki-67	High Ki-67		Low Ki-67	High Ki-67	
**Age (years)**	64.9 ± 9.0	66.6 ± 9.4	0.23	60.6 ± 9.6	67.3 ± 7.9	0.01	60.8 ± 9.5	65.9 ± 12.6	0.13
**Gender**			1.00			0.64			0.30
Male	49 (66.2)	28 (65.1)		19 (61.3)	13 (72.2)		12 (46.2)	5 (26.3)	
Female	25 (33.8)	15 (34.9)		12 (38.7)	5 (27.8)		14 (53.8)	14 (73.7)	
**Smoking status**			1.00			0.57			0.15
Never smoker	33 (44.6)	19 (44.2)		16 (51.6)	7 (38.9)		15 (57.7)	6 (31.6)	
Smoker	41 (55.4)	24 (55.8)		15 (48.4)	11 (61.1)		11 (42.3)	13 (68.4)	
**Pathological type**			0.01			0.09			<0.01
LUAD	62 (83.8)	27 (62.8)		27 (87.1)	12 (66.7)		23 (88.5)	9 (47.4)	
LUSC	12 (16.2)	16 (37.2)		4 (12.9)	6 (33.3)		3 (11.5)	10 (52.6)	
**Longest diameter (mm)**	30.1 ± 15.2	42.0 ± 15.0	<0.001	29.0 ± 12.6	45.2 ± 15.1	<0.001	28.5 ± 12.9	42.8 ± 17.3	<0.01
**Lobulation sign**			0.15			0.70			0.60
Absence	18 (24.3)	5 (11.6)		8 (25.8)	3 (16.7)		7 (26.9)	3 (15.8)	
Presence	56 (75.7)	38 (88.4)		23 (74.2)	15 (83.3)		19 (73.1)	16 (84.2)	
**Spiculation sign**			0.03			0.19			0.01
Absence	37 (50.0)	31 (72.1)		17 (54.8)	14 (77.8)		11 (42.3)	16 (84.2)	
Presence	37 (50.0)	12 (27.9)		14 (45.2)	2 (22.2)		15 (57.7)	3 (15.8)	
**Pleural indentation**			<0.01			0.03			0.11
Absence	17 (23.0)	22 (51.2)		11 (35.5)	13 (72.2)		9 (34.6)	12 (63.2)	
Presence	57 (77.0)	21 (48.8)		20 (64.5)	5 (27.8)		17 (65.4)	7 (36.8)	
**Liquefaction necrosis**			<0.01			0.04			0.04
Absence	63(85.1)	23 (53.5)		24 (77.4)	8 (44.4)		23 (88.5)	11 (57.9)	
Presence	11 (14.9)	20 (46.5)		7 (22.6)	10 (55.6)		3 (11.5)	8 (42.1)	

The differences were assessed with the Mann-Whitney-Wilcoxon test or Chi-Squared test; LUAD, lung adenocarcinoma; LUSC, Lung squamous carcinoma.

### 3.2 Consistency analysis

In the set of radiomics features within ROIs extracted by two radiologists, 30 nodules were randomly selected and segmented independently by one radiologist. One month later, another radiologist segmented these 30 nodules. Correlation analysis was performed on 1316 features extracted from these 30 nodules by inter-class correlation (ICC), and the consistency was considered good at ICC>0.80. The resultant ICC in this study was 0.84.

### 3.3 Selection of radiomics features and construction of the radiomics signature

**Figure 3:**
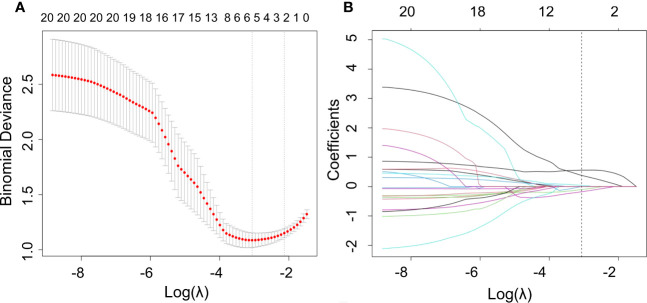
Optimal radiomics features were screened using mRMR and LASSO. **(A)** Binomial deviation versus a parameter. LASSO regression was used to screen the radiomics features, and cross-validation was used to select the optimal model parameter λ. The vertical axis was the binomial deviation, and the horizontal axis was the log (λ)value. λ, which represented the smallest binomial deviation of the model, was the optimal value (vertical dashed line). **(B)** Graph of the variation of the radiomics feature coefficient with λ. The number above indicates the number of filtered features.

**Figure 4:**
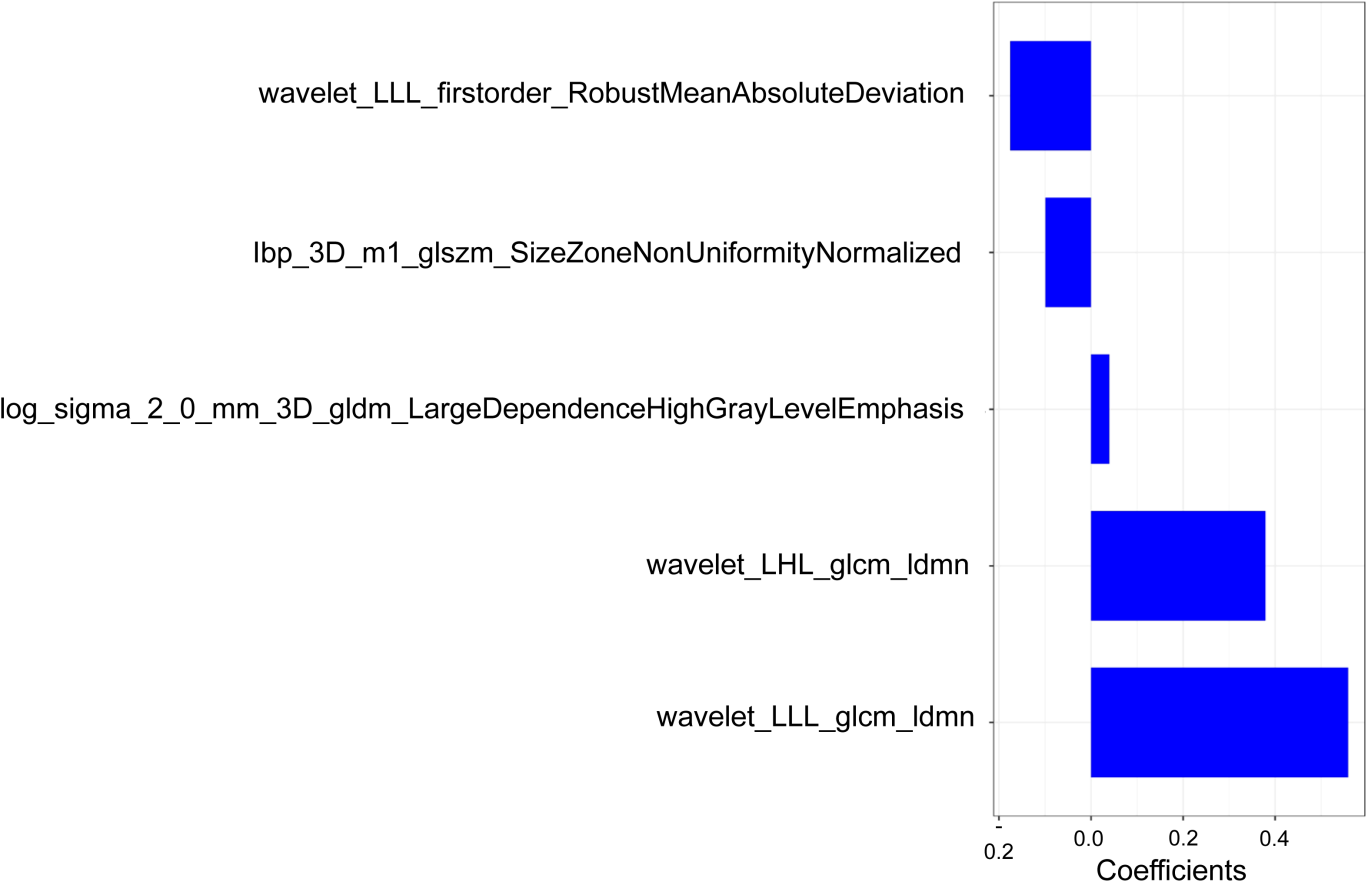
Optimal radiomics features used in this study and their corresponding weights.

**Figure 5:**
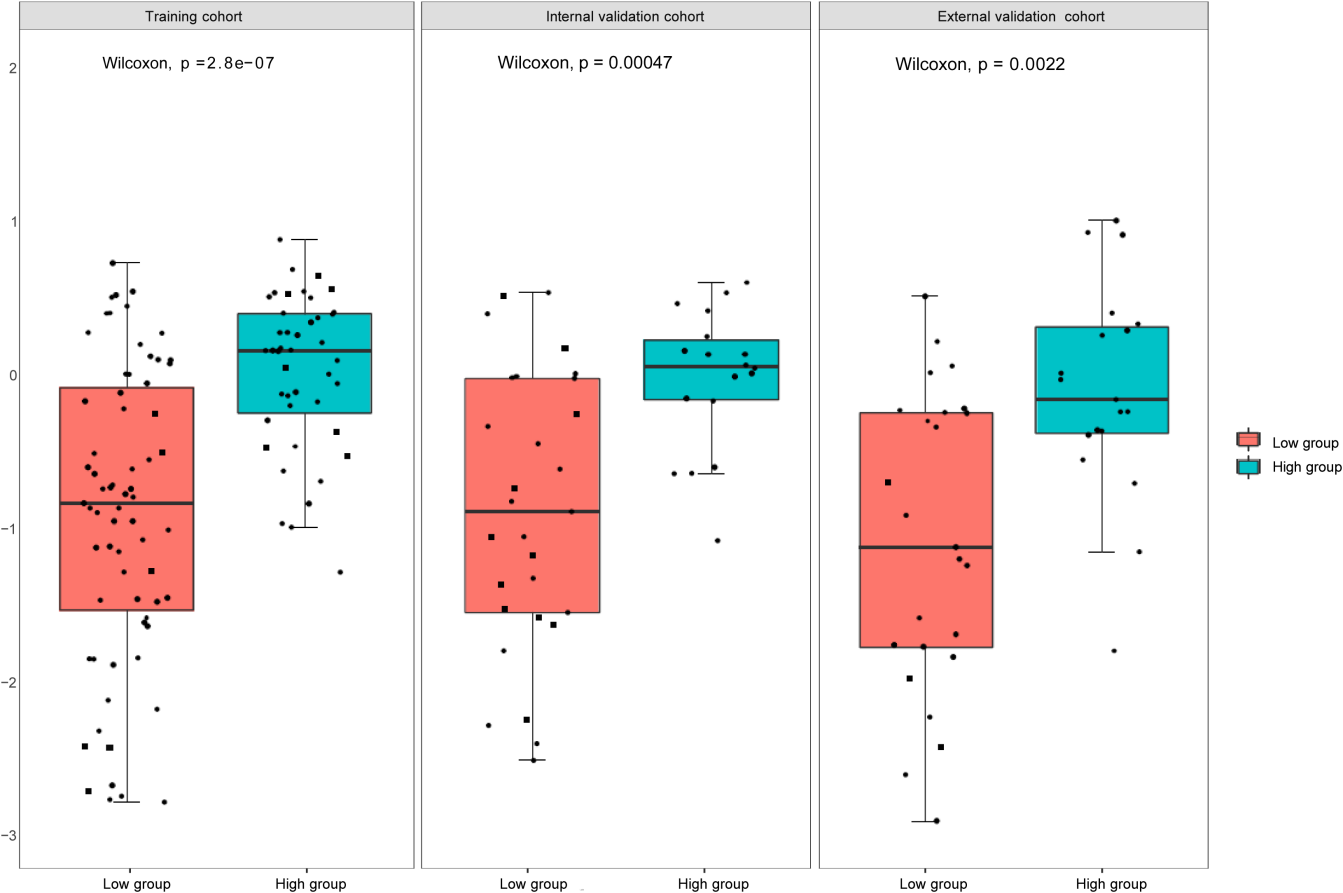
Rad -scores in the training, internal, and external validation cohorts. There is a significant difference between the rad- scores of the high and low expression groups (*P*<0.05).

We used the mRMR to select the feature with the strongest correlation. Finally, LASSO was utilized to choose the optimal features in constructing the radiomics signature, and five features were selected (**Figures 3**, **4**). Based on the final five radiomics features and their weights, the rad-score calculation formula was derived, and the rad-score value for each patient was calculated separately. The rad-scores of each patient across all three cohorts are shown in **Figure 5**.

### 3.4 Establishment of a nomogram combining radiomics and clinical risk factors

**Figure 6:**
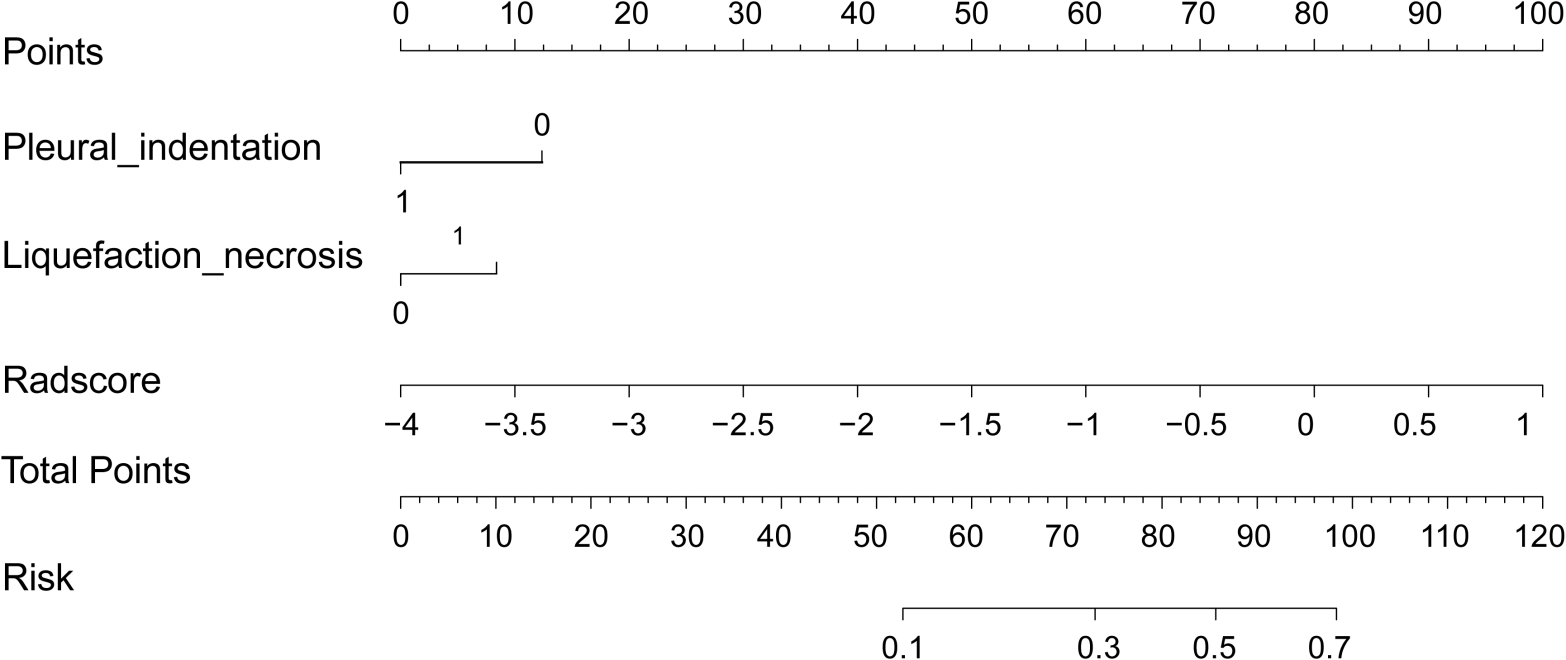
Nomogram integrating radiomics signatures and clinical features.

**Figure 7:**
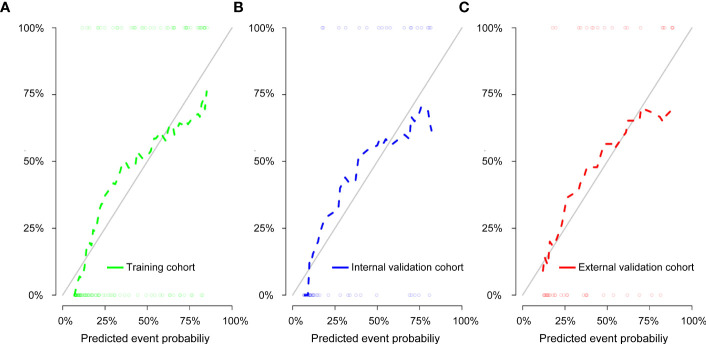
Calibration curve of the nomogram for the training cohort **(A)**, internal validation cohort **(B)**, and external validation cohort **(C)**. The solid diagonal line represents the perfect prediction of the ideal model, and the dashed line represents the actual model’s performance.

**Figure 8:**
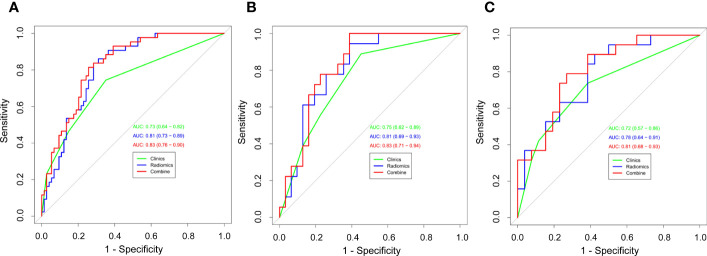
Receiver operating characteristic (ROC) curves of all three models were used to predict the Ki-67 expression level between the training cohort **(A)**, internal validation cohort **(B)**, and external validation cohort **(C)**.

We then performed a univariable analysis, which revealed that the longest diameter, spiculation sign, pleural indentation, and liquefaction necrosis are significantly associated with high expression of Ki-67 (*P*<0.05) (**Table 3**). Subsequent multivariable logistic regression analysis demonstrated that pleural indentation and liquefaction necrosis were independent predictors of the Ki-67 expression in pure-solid NSCLC (**Table 3**). Based on these two independent predictive factors, we established a clinical model related to the Ki-67 expression level. Logistic regression was used to establish a radiomics prediction model for Ki-67 expression levels in pure-solid NSCLC patients. Finally, a nomogram combining radiomics signature and clinical features was constructed (**Figure 6**). The calibration curve for the probability of Ki-67 expression levels showed that the nomogram matched well with the actual trend in all three cohorts (**Figure 7**). The DeLong test showed a significant difference between the AUC of the nomogram and the AUC of the clinical model in the training cohort (*P*<0.05). However, there was no statistical difference in AUC between the radiomics model and the nomogram (*P*=0.14). There was no significant statistical difference in AUC among the models in both internal and external validation cohorts (*P*>0.05). **Figure 8** and **Table 4** show that nomogram‘s prediction efficiency outperformed the radiomics signature and clinical model in all three study cohorts.

**Table 3:** Univariable and Multivariable Logistic Regression analyses in the training cohort.

Variable	Univariable Analysis		Multivariable Analysis	
	OR (95% CI)	*P*-value	OR (95% CI)	*P-*value
Gender	1.05 (0.48–2.32)	0.90	…	…
Age	1.03 (0.98–1.07)	0.23	…	…
Smoking status	1.02 (0.48–2.17)	0.97	…	…
Longest diameter	1.05 (1.02–1.08)	<0.001	…	…
Lobulation sign	2.44 (0.84–7.14)	0.10	…	…
Spiculation sign	0.39 (0.17–0.87)	0.02	…	…
Pleural indentation	0.28 (0.13–0.64)	0.002	0.27 (0.11–0.64)	0.003
Liquefaction necrosis	4.98 (2.07–11.97)	<0.001	5.25 (2.08–13.21)	<0.001

**Table 4:** The predictive values of different models across three cohorts of the study.

Models	AUC	95%CI	Accuracy	Sensitivity	Specificity	PPV	NPV
Training cohort							
Clinical Model	0.73	0.64–0.82	0.68	0.74	0.65	0.55	0.81
Radiomics Model	0.81	0.73–0.89	0.76	0.84	0.72	0.63	0.88
Combined Model	0.83	0.76–0.90	0.77	0.82	0.74	0.65	0.87
Internal validation cohort							
Clinical Model	0.75	0.62–0.89	0.67	0.89	0.55	0.53	0.89
Radiomics Model	0.81	0.69–0.93	0.69	0.78	0.64	0.56	0.83
Combined Model	0.83	0.71–0.94	0.73	0.83	0.68	0.60	0.88
External validation cohort							
Clinical Model	0.72	0.57–0.86	0.66	0.74	0.62	0.58	0.76
Radiomics Model	0.78	0.64–0.91	0.68	0.79	0.62	0.60	0.80
Combined Model	0.81	0.68–0.93	0.76	0.74	0.77	0.70	0.80

AUC, the area under the curve; CI, confidence interval; NPV, negative predictive value; PPV, positive predictive value.

### 3.5 Evaluation of the clinical value of the nomogram with DCA

**Figure 9:**
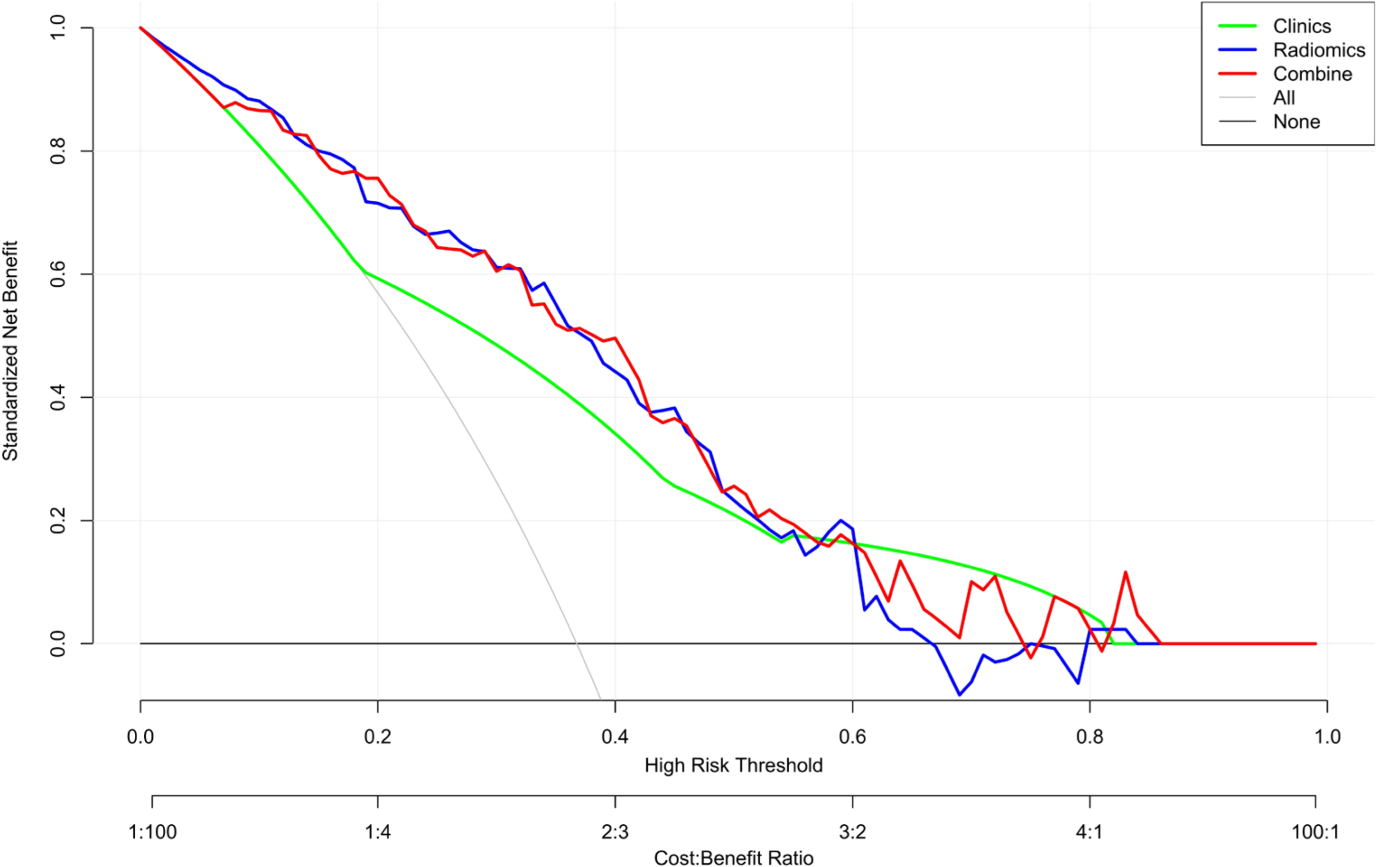
Decision curve analysis (DCA) of the nomogram. The y-axis represents the net benefit. The x-axis shows the threshold probability—the expected benefit of the number of treatments equals the expected benefit of not receiving treatment. The gray line represents the hypothesis that all patients have high Ki-67 expression, and the black line represents the hypothesis that no patient has high Ki-67 expression. The red curve is farthest from the X and Y axes, indicating that the nomogram is clinically useful.

The DCA was used to assess the clinical practical value of the nomogram in all three models (**Figure 9**). In this study, the DCA indicated that the net benefit of the nomogram combining radiomics signature and clinical features was higher than that of the clinical and radiomics models when the threshold probability was 0.07 to 0.60 and treated all or no patients.

## 4 Discussion

NSCLC is a malignancy with an exceptionally high mortality rate. Squamous and adenocarcinoma are the most common pathological types of NSCLC (1). Currently, Ki-67 is the most frequently employed marker for evaluating cell proliferation. Its expression strongly correlates with lung cancer development, metastasis, and prognosis (17). Accurately predicting Ki-67 expression levels may assist clinicians in making correct treatment decisions and customizing patient care.

Radiomics is the process of acquiring high-throughput data, extracting the quantitative features from images using computer learning software, mining quantitative information, and filtering out the most valuable radiological features to construct a predictive model (18, 19). Radiomics can be a better, non-invasive alternative to biopsy by improving treatment efficiency and reducing costs. Previous studies performed radiomics using images from positron emission tomography/computed tomography (PET/CT) and magnetic resonance imaging (MRI) (16, 20, 21). Many hospitals lack advanced imaging equipment, but CT is available in most of them. As a non-invasive, convenient, and quick imaging method, CT is a valuable imaging tool for lung cancer diagnosis and outcome evaluation. Because textural features of the original structure of the lesion tissue could be obscured by the high-density contrast in the enhanced CT images (22), only thin-slice CT non-enhanced images were used in this study. Since different radiological subtypes of lung cancer may have different biological behaviors, it is necessary to explore the value of radiomics in pure-solid and subsolid lung cancer separately. Few multicenter studies explored the expression level of Ki-67 in pure-solid lung cancer predicted by radiomics. Our multicenter study aimed to develop a simple, non-invasive, and widely applicable method to predict Ki-67 expression levels in solid NSCLC using thin-slice CT non-enhanced images. Thus, analyzing CT images of NSCLC may be valuable for predicting Ki-67 PI.

Several clinical and radiographic characteristics correlate with Ki-67 expression levels. According to previous studies, higher Ki-67 expression levels were linked to age, gender, and tumor diameter (8, 23). We observed significant differences in age between the Ki-67-high and Ki-67-low expression groups; in addition, all three cohorts showed significant differences in tumor diameter (*P*<0.05) but not in gender (*P>*0.05). This could be explained by including only NSCLC patients with pure-solid nodules or masses, not those with ground-glass nodules. In addition, logistic regression analysis showed that the radiographic sign (liquefaction necrosis) was an independent predictor of Ki­67 high expression in pure-solid NSCLC, and this may be explained by the fact that these radiographic signs always appear on CT scans of lesions containing fast-growing tumor cells, characterized by high Ki-67 levels. In necrocytosis, aqueous fluid within the lesion is thought to be caused by chronic ischemia and neovascularization associated with rapid tumor cell growth (24), often suggesting high heterogeneity within the tumor lesion. Therefore, the Ki-67 high-expression group is more likely to be more necrotic. We established a clinical model to predict the Ki-67 PI. The AUC values for the clinical model in the training, internal, and external validation cohorts were 0.73, 0.75, and 0.72, respectively. These data suggest that the clinical model may have a limited value in predicting Ki-67 expression levels, consistent with previous research (8).

The proliferation, differentiation, and cellular composition of subclonal regions of tumors with different Ki-67 expression levels may differ significantly (25). Medical imaging, such as CT, can reveal these subtle differences and present them as features (18); This study selected five optimal radiomics features and used them for establishing radiomics label values and a multivariable logistic regression model. Further testing of radiomics’ ability to predict Ki-67 expression in pure-solid NSCLC revealed that all three cohorts of radiomics models performed better than clinical models. This suggests that radiomics characteristics models might better predict Ki-67 expression levels in pure-solid NSCLC than prediction algorithms relying solely on clinical factors.

Additionally, we created a nomogram prediction model that combined radiomics signatures and clinical factors. The predictive power of the nomogram model was marginally greater than that of the radiomics model alone, but it outperformed the clinical model. Previous studies only used a single patient dataset and did not perform an external validation (13, 16).

In accordance with previous studies, a cutoff value of 40% was used for the Ki-67 expression in our study. However, other groups applied different cutoffs in their studies (12, 14, 26). Different pathological subtypes of lung cancer may exhibit varying levels of Ki-67 expression. Therefore, different Ki-67 cutoff values may be needed for different cases (27). This may explain the lack of an agreed-upon standard for the Ki-67 PI cutoff value. We intend to explore the predictive value of radiomics for Ki-67 expression using different Ki-67 cutoff values in our follow-up study.

There are several limitations in this study. First, this was a retrospective study, resulting in biased patient selection. Second, although it was a multicenter study with external validation, the sample size was relatively small. Our findings will need to be further validated using larger cohorts of patients. Finally, our study only included squamous cell carcinoma and adenocarcinoma, not other rare types of lung cancer, which may have resulted in some other biases.

## 5 Conclusion

The nomogram combining the radiomics signature and clinical features may be helpful, non-invasive, and effective for predicting Ki-67 expression levels in patients with pure-solid NSCLC.

## Data availability statement

The raw data supporting the conclusions of this article will be made available by the authors, without undue reservation.

## Ethics statement

The studies involving humans were approved by the institutional ethics committee of the Second Affiliated Hospital, Hengyang Medical School, University of South China. The studies were conducted in accordance with the local legislation and institutional requirements. The participants provided their written informed consent to participate in this study.

## Author contributions

FL: Funding acquisition, Investigation, Writing-original draft, Writing. QL: Investigation, Writing-original draft, Writing. XF: Conceptualization, Investigation, Writing – review & editing, Supervision. QY: Conceptualization, Investigation, Writing – review & editing, Supervision. ZX: Data curation, Investigation. YZ: Data curation, Investigation. XL: Investigation, Writing – review & editing. FTL: Data curation, Resources. YH: Data curation, Resources. HL: Data curation, Resources. All authors contributed to the article and approved the submitted version
